# The Intensity of Victimization: Associations with Children's Psychosocial Well-Being and Social Standing in the Classroom

**DOI:** 10.1371/journal.pone.0141490

**Published:** 2015-10-29

**Authors:** Rozemarijn van der Ploeg, Christian Steglich, Christina Salmivalli, René Veenstra

**Affiliations:** 1 Department of Sociology, University of Groningen, Groningen, the Netherlands; 2 Department of Psychology, University of Turku, Turku, Finland; University of Stellenbosch, SOUTH AFRICA

## Abstract

The association between experienced victimization and students' psychological and social adjustment depends on the intensity of victimization. We examined how *frequency* and *multiplicity* of victimization, and *the number of bullies involved*, account for differences in students’ psychosocial well-being and social standing in the classroom. Multilevel analyses were conducted on the control group of an intervention study among students in grades 3–6 of Dutch elementary schools (*N* = 2859 students from 124 classes and 33 schools; ages 8–12; 49.6% boys). It was found that victims of frequent and multiple victimization, and victims who were victimized by several bullies, had higher levels of psychosocial adjustment problems than victims of less frequent and non-multiple victimization, and victims with only one bully. Moreover, these more severe victims turned out to be least accepted and most rejected among their classmates. The findings illustrate that it can be fruitful to use several measures of victimization so that (differences in) adjustment problems can be better understood. Moreover, the results suggest that it is important to find out who is victimized, in what ways, and by whom. Anti-bullying interventions should provide resources to do this.

## Introduction

School bullying is a widespread problem. All over the world, large numbers of children are victimized by their peers [1]. Bullying is commonly defined as repetitive and intentional negative behavior against a victim who finds it difficult to defend himself or herself [2]. From previous research we know that victimization is related to various forms of psychosocial maladjustment [3–5]. Victims of bullying are often frightened to go to school, suffer from low self-esteem, and are more likely to be anxious or depressed[6–9]. Moreover, victims tend to be isolated and generally have a low social standing in the classroom [10,11]. While strong evidence has been found for the negative consequences of being victimized, various studies have shown that the emergence of psychological and social adjustment problems varies between victims [12,13].

In order to explain these differences in the level of victims' psychosocial well-being, researchers have investigated in which contexts suffering from victimization results in more severe maladjustment [14,15]. The focus was primarily on the broader social context in which the victimization takes place, such as the classroom [1,16–18]. However, it can be argued that, in addition to the classroom context, specific aspects of victimization itself account for differences in the level of students' psychosocial maladjustment and social standing in the classroom [19]. Researchers have recently recommended using measures of victimization that include other indicators of bullying besides frequency in order to better assess the harmfulness of bullying [20]: for instance, by how many peers and in how many ways the harassment is performed. Bullying behavior can be subdivided into several types, such as physical bullying (hitting, kicking), relational bullying (ignoring, gossiping), verbal bullying (calling names, insulting), material bullying (stealing or damaging things), and cyber bullying (via email or mobile phone). Being victimized through various types of bullying, e.g., being called names *and* being hit *and* being ignored, can be considered more intense than being victimized in one way, especially when the diverse bullying behavior is performed by the same peer. Victimization can also be experienced as more intense when it is performed by more than one peer, and when it happens frequently. In other words, three aspects of intensity can be distinguished: frequency, multiplicity, and the number of bullies involved.

Relatively few researchers who investigated associations between psychosocial maladjustment, social status, and victimization also addressed in how many ways (e.g., [21]) or by how many peers (e.g., [15]) children were victimized. The frequency of victimization has more often been taken into account (e.g., [10,22–24]), but often these specific aspects were neglected and just a distinction between victims and non-victims is made [8,17,25,26].

In line with Volk et al. [20], we argue that in order to better understand (differences in) the harmfulness of victimization, it can be important to take into consideration not only the frequency of victimization, but also the multiplicity of victimization and the number of bullies involved. Hence, the main goal of the present study was to examine how these three characteristics of victimization account for differences in several psychosocial outcomes. More specifically, we took a closer look at students’ psychosocial well-being (i.e., social anxiety, depressive symptoms, and well-being at school) and social standing in the classroom (i.e., acceptance, rejection, and perceived popularity). For explorative reasons we also included gender and gender interactions in our analyses, as differences may exist between boys and girls in psychosocial well-being, social standing, and reactions to victimization [3,25,27].

## Theoretical Background

### Victimization and Students’ Psychosocial Well-Being

Being victimized may lead to psychosocial adjustment problems when children feel that they deviate from their peer group. This can be explained by attributional processes [28,29]. Attribution theory is concerned with the perceptions people have of why a certain (negative) event has taken place, referring to how they rationalize or make sense of it. Victims, for instance, may wonder why they are victimized. In the wide range of potential answers to this question, three dimensions are generally distinguished [6,28]: stability (whether the perceived cause of victimization is stable or varies over time), controllability (whether the perceived cause of victimization can be altered by the victim), and locus (whether the cause of victimization is internal or external to the victim). These dimensions are considered to be related to victims’ psychosocial adjustment [29].

In sum, attribution theory can be used as a heuristic to better understand under which conditions—the frequency and multiplicity of victimization, and the number of bullies involved—experiencing victimization might lead to worse psychosocial outcomes.

In the current study the internal locus was of specific interest because particularly self-blaming attributions characterize how victims interpret harassment by peers [6]. A negative event can be internally evaluated in two ways: characterological and behavioral self-blame [30]. Characterological self-blame refers to the tendency to attribute negative events to stable and uncontrollable features of the self: “*It is something about the way I am”*. Behavioral self-blame, in contrast, is the tendency to attribute events to unstable and controllable features of the situation, such as one's own behavior: *“It is something about what I did*” [6,28]. It can be argued that maladaptive outcomes are particularly present among victims who attribute the harassment to personal characteristics rather than their behavior. These victims may feel that they lack control over the situation and, hence, be more likely to expect the victimization to happen again [6,19]. Indeed, Boulton [31] found that the association between childhood social exclusion, verbal victimization, and relational victimization, on the one hand, and adult social anxiety, on the other hand, was moderated by characterological self-blaming attributions.

When it comes to the specific aspects of victimization, it can be assumed that more frequent harassment, victimization in multiple ways, and victimization by several bullies is predominantly associated with characterological self-blame. For instance, it was found that the more frequently victimization takes place, the more likely it is that victims will feel that they are unable to stop peers from victimizing them [19]. Frequent victimization is then likely to be attributed to personal characteristics, such as incapability to stand up for oneself. Moreover, Nishina [15] argued that victims’ expectations of future victimization increase when the harassment is performed by several bullies, given that they are the target of a broader range of peers and not just random victims. In others words, victims tend to perceive the victimization as uncontrollable and stable when more than one bully is involved and, hence, are more likely to assign the victimization to features of the self. Lastly, being victimized in multiple ways (e.g., being called names, being hit, and being ignored] by the same peer sends a message to victims that they are not random recipients of aggression but rather *the target* of bullies, making it less likely to assign the victimization to the situation.

In sum, when the bullying behavior is more intense (i.e., more frequent, performed in multiple ways, or by more than one bully), the harassment is more likely to be attributed to personal characteristics, than to features of the situation, resulting in higher levels of psychosocial maladjustment. We expected psychosocial well-being to be lower for frequently victimized students (*Hypothesis 1*), students who are victimized in multiple ways by the same peer (*Hypothesis 2*), and victims with several bullies (*Hypothesis 3*).

### Victimization and Social Standing in the Classroom

Social standing in the peer group is an important aspect of (early) adolescent life [32,33]. Especially in schools, students tend to form social hierarchies in which likeability and visibility play an important role [34]. When it comes to social status, a distinction between "likeability" and "perceived popularity" is usually made. Likeability refers to the degree to which someone is accepted (liked] versus rejected (disliked] by peers. Popularity refers more to dominance, status, and visibility in the peer group [35,36]. Popular peers are the ones with whom many want to spend time or associate with [33].

From a range of previous research findings we know that being victimized is associated with social standing in the classroom. It has repeatedly been found that victims generally score high on social rejection and low on social acceptance [11,37] and are perceived as less popular [10,38]. The low status of victims in the peer group can be understood both as a cause and a consequence of victimization. Low social standing can be considered a reason for being victimized, as previous studies have suggested that most bullies tend to choose so-called “easy targets” to harass: that is, peers who are disliked, or perceived as unimportant by others [1,34]. However, it has also been argued that peers’ perceptions of victims change gradually when the victimization endures and becomes more apparent. If the harassment persists and its visibility increases, victims may be seen as more worthless or risky to associate with, as a result of which their likeability and popularity in the peer group decreases [11,39,40].

Based on the latter, it can be assumed that the negative consequences with regard to social standing in the classroom depend on the frequency and multiplicity of the harassment, as well as the number of bullies involved. After all, the victimization becomes more visible when it occurs more frequently, happens in multiple ways, and is performed by several peers. This greater visibility of the victim is likely to result in greater social rejection by classmates and a less prominent position in the peer group, leading to a lower popular status. Although we were unable to examine causal direction in the current study, we expected that the social standing of victims who were frequently victimized would be lower than that of victims who were occasionally or not at all victimized (*Hypothesis 4*). Also the social standing of victims who were victimized in multiple ways was expected to be lower than that of victims who were victimized in only one way by the same peer (*Hypothesis 5*). Lastly, we hypothesized that the social standing of victims in the classroom would decrease as the number of bullies increased *(Hypothesis 6)*.

## Method

### Sample

Data used in this study stem from the evaluation of the Dutch implementation of the KiVa anti-bullying program. To recruit schools, letters describing the KiVa project were sent in the fall of 2011 to all 6,938 Dutch elementary schools. Special elementary schools and schools for children with special educational needs could not take part in the KiVa program and were hence not invited to participate. A total of 99 schools indicated that they were willing to participate.

The schools were randomly assigned by the Netherlands Bureau for Economic Policy Analysis (CPB) to either the control condition (33 schools) or one of the two intervention conditions: KiVa (34 schools) or KiVa+ (32 schools). KiVa+ is the Finnish KiVa program with one additional component. Teachers in KiVa+ schools receive reports about the social structure of their classroom. Control schools were asked to continue their “care as usual” anti-bullying approach until their participation in the KiVa program in June 2014.

### Procedure

Two times per school year (October and May) students filled in internet-based questionnaires in the schools’ computer labs during regular school hours. Classroom teachers distributed individual passwords to their students, which could be used to access the questionnaire. Students read all questions by themselves; difficult topics were explained in instructional videos. In these videos, a professional actress explained the questions in such a way that all students would understand them (e.g., by talking slowly and articulating words clearly). The term *bullying* was defined in the way formulated in Olweus’ Bully/Victim questionnaire [41]. Several examples covering different forms of bullying were given, followed by an explanation emphasizing the intentional and repetitive nature of bullying and the power imbalance. Classroom teachers were present to answer questions and to assist students when necessary. Teachers were supplied with detailed instructions before the data collection started, and were encouraged to help students in such a way that it would not affect their answers (e.g., by asking them questions such as “Which words are unclear to you?”). The order of questions and scales used in this study were randomized in such a way that the order of presentation of the questions would not have any systematic effect on the results.

### Ethics statement

Two of the authors were responsible for the data collection. Observational research using data does not fall within the ambit of the Dutch Act on research on human subjects and does not need approval of an ethics committee. The data were anonymized before the analyses, and questionnaires were completed on a voluntary basis. Schools sent information about the study and permission forms to parents. Parents who did not want their child to participate in the assessment were asked to return the form. Students were informed at school about the research and gave oral consent. Both parents and students could withdraw from participation at any time. Students who did not receive parental consent, or who did not want to participate themselves, or who were unable to fill in the questionnaire, did not participate (1.5%). The main reason for this high response rate is that data were collected online and teachers were informed about which of their students filled in the questionnaire. Moreover, students who incidentally missed the scheduled day of data collection could participate on another day within a month.

### Participants

In the present study, we used data from schools in the control condition that were collected in October 2012. The focus of our study was on victimization within the classroom. However, some students reported that they were victimized only by peers from other classrooms or other schools (20%). These students were also included in our analyses through addition of a dummy variable that indicated whether or not students were only bullied outside the classroom. In total, 3.8% of the students had missing data on one of our study variables. They were excluded from the sample. The remaining sample consisted of 33 schools, 124 classrooms, and 2859 students in grades 3–6 (ages 8–12; Dutch grades: 5–8; 49.6% boys).

### Measures

#### Psychosocial well-being

Students' psychosocial well-being was indicated by their levels of social anxiety, depressive symptoms, and well-being at school. We used a seven-item scale, derived from the Social Phobia Screening Questionnaire [42], to measure *social anxiety*. Students responded on a five-point scale to items such as “I am scared to be together with others during the break” (1 = never, 5 = always). The scores for the seven items formed an internally consistent scale and were averaged (Cronbach’s α = .77).

To measure the emergence of *depressive symptoms*, nine items from the Major Depression Disorder Scale [43] were used (e.g., “I feel worthless”). Students’ answers could vary from never (1) to always (1). Together, the items formed an internally consistent scale and were averaged (Cronbach’s α = .81).

*Well-being at school* was indicated by seven items concerning perceptions of the classroom and school [44]. Students responded to items such as “I feel I am accepted as I am at school” (1 = never, 4 = always). The items formed an internally consistent scale (Cronbach’s α = .83) and were averaged.

#### Social standing in the classroom

The social standing of students in the classroom was determined using their acceptance and rejection, on the one hand, and perceived popularity, on the other hand. *Acceptance* and *rejection* were measured by asking students to nominate an unlimited number of classmates they liked most (acceptance) and liked least (rejection). To assess *perceived popularity*, participants nominated an unlimited number of classmates they perceived as most popular (“Which of your classmates is popular?”). For each student, the received nominations for "being liked", “being disliked”, and "popularity" were summed and divided by the number of nominating classmates so that proportion scores for, respectively, acceptance, rejection, and perceived popularity were created (0–1).

#### Characteristics of victimization

In order to conduct the analyses, we constructed both a categorical and a continuous measure for the indicators of victimization. *Frequency of victimization* was measured using the revised Olweus Bully/Victim questionnaire [41]. Students had to indicate how many times they had been victimized in the past months. They could answer on a five-point scale (1: it did not happen, 2: once or twice, 3: two or three times a month, 4: about once a week, 5: several times a week). In accordance with the recommendation of [45], students who indicated being victimized at least two or three times a month were considered to be often victimized.

The measure of *multiplicity of victimization* was also based on self-reports. Students were asked to indicate by which classmates they were victimized. Five forms of victimization were distinguished: physical (hitting, kicking), relational (ignoring, gossiping), verbal (calling names, insulting), material (stealing or damaging things), and digital (cyber bullying). Moreover, students could specify which classmates initiated the bullying. Students who nominated the same peer for at least three out of these six questions were considered to be victimized in multiple ways by the same peer. When students nominated more than one peer at least three times, they were considered to be multiply victimized by several peers. A continuous measure of multiplicity was generated by dividing for each student the sum of the reported nominations by the total number of bullies involved. Correlations between the various forms of victimization were all positive (ranging from .21 to .57) and significant (*p* < .001).

Lastly, each student could indicate by which classmates they were victimized; using this information, we created a measure of *the number of bullies* a victim has. Table 1 presents descriptive information on the study variables.

**Table 1:** Descriptive Information on the Study Variables (N = 2859).

	*Min*	*Max*	*Mean / %*	*SD*	*%*
Sex (1 = boy)	0	1	.50	.50	
Social anxiety	1	5	1.88	0.69	
Depressive symptoms	1	4	1.66	0.51	
Well-being at school	1	4	3.06	0.55	
Acceptance	0	.93	.41	.17	
Rejection	0	.96	.14	.14	
Perceived popularity	0	.90	.13	.16	
Frequency of victimization (continuous)	0	4	0.93	1.30	
Frequency of victimization (categorical)					
Not victimized					53.4%
Sometimes victimized					25.0%
Often victimized					21.6%
Multiplicity of victimization (continuous)	0	6	0.58	0.97	
Multiplicity of victimization (categorical)					
Not victimized					53.4%
Victimized in one way by classmate					34.8%
Victimized in multiple ways by one classmate					5.1%
Victimized in multiple ways by several classmates					6.7%
Number of bullies (continuous)	0	27	1.29	2.83	
Number of bullies (categorical)					
Not victimized					53.4%
Victimized outside the classroom					20.4%
One bully in classroom					5.8%
Several bullies in classroom					20.4%

### Analyses

We first examined whether the means in psychosocial well-being and social standing in the classroom differed as a result of the frequency and multiplicity of victimization, and the number of bullies involved, using analyses of variance (ANOVA). The results are shown in Tables 2, 3 and 4.

**Table 2:** Psychosocial Well-being and Social Standing by Frequency of Victimization.

	Not victimized		Sometimes victimized		Often victimized		η^2^
	*Mean*	*SD*	*Mean*	*SD*	*Mean*	*SD*	
Social anxiety	1.78^a^	0.64	1.91^b^	0.65	2.09^c^	0.85	.030
Depressive symptoms	1.53^a^	0.44	1.70^b^	0.45	1.94^c^	0.59	.102
Well-being at school	3.19^c^	0.51	2.98^b^	0.53	2.82^a^	0.59	.078
Acceptance	.44^c^	.16	.41^b^	.17	.37^a^	.17	.027
Rejection	.11^a^	.13	.14^b^	.14	.21^c^	.17	.071
Perceived popularity	.14^b^	.17	.13^b^	.17	.09^a^	.12	.014
*N*	1526		716		617		

Note.

^a, b, c^ *Differences in means are significant at* .*05 level*.

**Table 3:** Psychosocial Well-being and Social Standing by Multiplicity of Victimization.

	Not Victimized		Victimized outside the classroom		Victimized in one way by classmate(s)		Victimized in multiple ways by one classmate		Victimized in multiple ways by several classmates		η^2^
	*Mean*	*SD*	*Mean*	*SD*	*Mean*	*SD*	*Mean*	*SD*	*Mean*	*SD*	
Social anxiety	1.78^a^	0.64	1.94^b^	0.70	1.96^b^ ^b^	0.71	2.06^c^ ^b^	0.79	2.17^c^	0.85	.029
Depressive symptoms	1.53^a^	0.44	1.79^b^	0.54	1.74^b^ ^b^	0.47	1.82^b^ ^b^	0.52	2.01^c^	0.59	.089
Well-being at school	3.19^c^	0.51	2.99^b^	0.54	2.92^a^ ^b^	0.53	2.78^a^ ^b^	0.57	2.72^a^	0.63	.082
Acceptance	.44^c^	.16	.41^b^	.16	.39^b^ ^b^	.17	.38^a^ ^b^	.18	.33^a^	.17	.031
Rejection	.11^a^	.13	.16^b^	.15	.16^b^ ^b^	.14	.19^b^ ^b^	.16	.25^c^	.19	.070
Perceived popularity	.14^b^	.17	.13^b^	.16	.10^a^ ^b^	.15	.11^a^	.16	.08^a^	.12	.013
*N*	1526		583		414		145		191		

Note.

^a, b, c^ *Differences in means are significant at* .*05 level*.

**Table 4:** Psychosocial Well-being and Social Standing by Number of Bullies Involved.

	Not victimized		Victimized outside the classroom		One bullyinvolved		Several bullies involved		η^2^
	*Mean*	*SD*	*Mean*	*SD*	*Mean*	*SD*	*Mean*	*SD*	
Social anxiety	1.78^a^	0.64	1.94^b^ ^c^	0.70	1.91^a^ ^b^	0.71	2.07^c^	0.77	.028
Depressive symptoms	1.53^a^	0.44	1.79^b^ ^c^	0.54	1.73^b^ ^c^	0.47	1.85^b^	0.54	.079
Well-being at school	3.19^c^	0.51	2.99^b^ ^c^	0.54	2.91^a^ ^b^	0.56	2.83^a^	0.57	.076
Acceptance	.44^c^	.16	.41^b^ ^c^	.16	.39^a^ ^b^	.16	.37^a^	.18	.025
Rejection	.11^a^	.13	.16^b^ ^c^	.15	.16^b^ ^c^	.15	.20^c^	.17	.056
Perceived popularity	.14^c^	.17	.13^b^ ^c^	.16	.10^a^ ^b^	.14	.10^a^	.14	.012
*N*	1526		583		167		583		

Note.

^a, b, c^ *Differences in means are significant at* .*05 level*

Our hypotheses were tested using multilevel regression techniques [46], with students nested in classrooms in schools. All models were estimated using Stata 13. In order to facilitate the interpretation of the outcomes, all continuous variables were standardized across the whole sample (*M* = 0, *SD* = 1). To investigate the additional value of multiplicity of victimization and the number of bullies involved, the effects of frequency of victimization were examined first (Models A). Subsequently, indicators of multiplicity and the number of bullies were added (Models B). The results are presented in Tables 5 and 6.

**Table 5:** Multilevel Regression Analyses: Effects of Victimization on Psychosocial Well-being (N = 2859).

	Social anxiety				Depressive symptoms				Well-being at school			
	*Model 1a*		*Model 1b*		*Model 2a*		*Model 2b*		*Model 3a*		*Model 3b*	
	*B*	*SE*	*B*	*SE*	*B*	*SE*	*B*	*SE*	*B*	*SE*	*B*	*SE*
Intercept	0.16	0.03	0.14	0.03	0.01	0.04	-0.04	0.03	0.06	0.04	0.10	0.04
Sex (1 = boy)	-0.36	0.04**	-0.36	0.04**	-0.02	0.04	-0.02	0.04*	-0.12	0.04**	-0.14	0.03**
Frequency of victimization	0.17	0.02**	0.12	0.03**	0.33	0.02**	0.24	0.03**	-0.26	0.02**	-0.15	0.03**
Multiplicity of victimization			0.03	0.03			0.04	0.03			-0.08	0.03*
Number of bullies			0.06	0.02*			0.08	0.02**			-0.13	0.02**
Victimized outside the classroom			0.07	0.06			0.21	0.06**			-0.17	0.06**
*Variance school level*	0.01	0.00	0.01	0.01	0.01	0.01	0.01	0.00	0.01	0.01	0.01	0.01
*Variance classroom level*	0.00	0.01	0.00	0.01	0.01	0.01	0.01	0.01	0.05	0.01**	0.05	0.01**
*Variance individual level*	0.92	0.02**	0.92	0.02**	0.87	0.03**	0.86	0.02**	0.87	0.02**	0.85	0.02**
*Decrease in deviance*	45 (*df* = 1)**		6 (*df* = 3)		162 (*df* = 1)**		12 (*df* = 3)*		106 (*df* = 1)**		31 (*df* = 3)**	

Decrease in deviance with the former model. The comparison in Model A is with the model in which only sex is included

** = p <.001;

* = p <.01

All variables (except sex and no bullies in classroom) were standardized.

**Table 6:** Multilevel Regression Analyses: Effects of Victimization on Social Standing in the Classroom (N = 2859).

	Acceptance				Rejection				Perceived Popularity			
	*Model 1a*		*Model 1b*		*Model 5a*		*Model 5b*		*Model 6a*		*Model 6b*	
	*B*	*SE*	*B*	*SE*	*B*	*SE*	*B*	*SE*	*B*	*SE*	*B*	*SE*
Intercept	0.15	0.07	0.16	0.07	-0.20	0.03	-0.22	0.03	-0.08	0.03	-0.10	0.04**
Sex (1 = boy)	-0.16	0.03**	-0.17	0.03**	0.40	0.03**	0.42	0.03**	0.20	0.04**	0.19	0.04**
Frequency of victimization	-0.20	0.02**	-0.14	0.03**	0.26	0.02**	0.16	0.03**	-0.13	0.02**	-0.12	0.03**
Multiplicity of victimization			-0.05	0.02			0.07	0.03*			-0.01	0.03
Number of bullies			-0.08	0.02**			0.11	0.02**			-0.03	0.02
Victimized outside the classroom			-0.02	0.05			0.08	0.06			0.10	0.06
*Variance school level*	0.06	0.04	0.06	0.04	0.00	0.00	0.00	0.00	0.00	0.00	0.00	0.00
*Variance classroom level*	0.26	0.04**	0.25	0.04**	0.07	0.01**	0.06	0.01**	0.07	0.01**	0.07	0.01**
*Variance individual level*	0.67	0.02**	0.66	0.02**	0.81	0.02**	0.80	0.02**	0.91	0.02**	0.91	0.02**
*Decrease in deviance*	75 (*df* = 1)**		18 (*df* = 3)**		103 (*df* = 1)**		26 (*df* = 3)**		24 (*df* = 1)**		5 (*df* = 3)	

Note. Decrease in deviance with the former model. The comparison in Model A is with the model in which only sex is included.

** = p <.001;

* = p <.01

All variables (except sex and no bullies in classroom) were standardized.

## Results

### Differences in Students’ Psychosocial Well-Being and Social Standing in the Classroom

It can be seen in Table 2 that students who were often victimized suffered from higher levels of social anxiety and symptoms of depressiveness than students who were sometimes or not at all victimized. Additionally, it is shown that they had the lowest well-being at school. Significant differences were also found in social standing in the classroom. Frequently victimized students were more rejected, less accepted, and perceived as less popular among their classmates than less frequently victimized students or non-victims.

Table 3 shows that when the victimization was performed in multiple ways, students were more socially anxious and had a lower level of well-being at school. Moreover, victims of multiple victimization performed by more than one classmate showed significantly higher levels of depressive symptoms than other victims and non-victims. Multiple victimization was also found to be related to a lower social standing in the classroom. Victims who were victimized in various ways by several bullies were the most rejected among their classmates, and significantly less accepted than victims of single victimization and non-victims. As regards popularity, it can be seen that those victims who were victimized by their classmates, whether in one way or in multiple ways, were perceived as less popular than victims who were victimized outside the classroom and non-victims.

In Table 4, outcomes on psychosocial well-being and social standing in the classroom are distinguished by the number of bullies a victim has. Victims who had several bullies in their classroom showed the highest levels of social anxiety. Moreover, their well-being at school was lower than that of victims with no bullies in the classroom. Almost no significant differences in social standing were found. Victims with several bullies in the classroom were only found to be more rejected by their classmates than those with one bully. Nevertheless, victims were less accepted and popular than non-victims. It thus seems that for being liked or perceived as popular, whether or not one is victimized is more important than the number of bullies one has.

### Victimization, Psychosocial Well-Being, and Social Standing in the Classroom

In Table 5 it can be seen that the frequency of victimization was associated with students’ psychosocial well-being. Students who were more frequently victimized scored significantly higher on social anxiety (*B* = 0.17) and depressive symptoms (*B* = 0.33), and showed a lower level of well-being at school (*B* = -0.26). The same pattern was found for students who had more than one bully in the classroom (Models B). These results are in line with Hypotheses 1 and 3, in which we expected psychosocial well-being to be lower for students who were, respectively, more frequently victimized or victimized by several bullies. However, concerning the multiplicity of victimization (*Hypothesis 2)*, only the association with well-being at school (*B* = -0.08) reached significance. Additionally, it appears that students who were victimized by non-classmates (peers outside the classroom) also had a lower level of psychosocial well-being, as they reported more symptoms of depression and a lower level of well-being at school.

Models A in Table 6 show that students who were more often victimized were less accepted (*B* = -0.20) and perceived as less popular (*B* = -0.13) by their classmates. In addition, they were more rejected (*B* = 0.26). These results are consistent with Hypothesis 4, in which we expected an increase in the frequency of victimization to be associated with a lower social standing in the classroom. Multiplicity of victimization was found to be associated with acceptance (*B* = -0.05) and rejection (*B* = 0.07). Victims with more than one bully in the classroom scored lower on acceptance among classmates (*B* = -0.08) and higher on rejection (*B* = 0.11) (see Models B). Hence, in terms of acceptance and rejection, the outcomes are in line with Hypotheses 5 and 6. For perceived popularity among classmates, though, no evidence for these hypotheses was found.

All in all, the findings show that the frequency of victimization was most strongly associated with the indicators of students’ psychosocial well-being and social standing in the classroom. However, when we added measures of multiplicity of victimization and the number of bullies involved, the fit of our models concerning depressiveness symptoms, well-being at school, acceptance, and rejection improved significantly.

### Differences between Boys and Girls

We also tested whether the effects of frequency and multiplicity of victimization, and the number of bullies involved, differed between boys and girls. It was found that multiplicity of victimization was associated with symptoms of depression for girls (*B* = 0.08, *t(2859)* = 2.29, *p* = .02), but not for boys (*B* = 0.00. *t(2859)* = 0.02, *p* = .98). In contrast, a higher number of bullies was associated with symptoms of depression for boys (*B* = 0.15 *t(2859)* = 4.15, *p* < .001), but not for girls (*B* = 0.03, *t(2859)* = 1.04, *p* = .30); the same pattern was found for social anxiety (boys: *B* = 0.11 *t(2859)* = 2.91, *p* = .004; girls: *B* = 0.03 *t(2859)* = 0.80, *p* = .42). The frequency of victimization was more strongly associated with social anxiety for girls (*B* = 0.16 *t(2859)* = 4.73, *p* < .001) than for boys (*B* = 0.08 *t(2859)* = 2.52, *p* = .01). Finally, gender differences in the association between the number of bullies and rejection were found. A higher number of bullies was more strongly associated with rejection among classmates for boys (*B* = 0.28 *t(2859)* = 5.03, *p* < .001) than for girls (*B* = 0.07 *t(2859)* = 2.49, *p* = .01).

## Discussion

The aim of this study was a thorough investigation of differences in the correlates of experiencing victimization, using a more comprehensive concept of victimization. In previous research on victimization, specific aspects of the victimization itself were often neglected; hence, a distinction between victims and non-victims was usually made [8,17,25,26]. Some studies took into account the frequency with which the victimization occurred: it was found that more frequent victimization is associated with higher levels of loneliness [23], depression [9,24], and suicidal ideation [22], as well as with a lower social standing in the peer group [10]. We argued that, in addition to the frequency of victimization, it might be important to also consider in how many ways and by how many peers a person is victimized [14,15,20,21]. Hence, we examined to what extent the frequency and multiplicity of victimization, and the number of bullies involved, were associated with (different levels of) students' psychosocial well-being and social standing in the classroom.

In line with previous research [3,4], we found that victimization is associated with greater psychosocial problems. In our study, victims showed higher levels of social anxiety and symptoms of depression and felt less comfortable at school. However, in the present study we also aimed to take the intensity of the victimization into consideration. We proposed that psychosocial adjustment problems would particularly emerge when the victimization happened more often, was performed in various ways, or was performed by more than one peer. Our findings demonstrate that victims of more frequent victimization and victims with several bullies were indeed more likely to show more symptoms of social anxiety and depressiveness, and to feel less comfortable at school. Especially the findings concerning the number of bullies involved contributes to our knowledge that being victimized by several bullies is not only associated with increased daily humiliation [15], but also with students’ psychological adjustment. Multiplicity of victimization appeared to be important only for students’ adjustment at school, as it was only related to a lower level of well-being at school.

We also hypothesized that students’ social standing in the classroom would be associated with the frequency and multiplicity of victimization, and the number of bullies involved. We found that frequently victimized students were less accepted, more rejected, and perceived as less popular among their classmates. Additionally, we found that victims who were victimized in various ways or by several bullies were less accepted and more rejected among their classmates than victims of non-multiple victimization and victims with one bully. In contrast, no significant association with a popular status was found. Thus, for being perceived as popular among classmates, it appears less important in how many ways and by how many people a person is victimized. These findings give nuance to previous findings that being victimized is associated with a lower social standing in the peer group [10,11,37,38].

With regard to gender differences, this study reveals that the number of bullies involved is especially important to boys’ psychosocial well-being and rejection by classmates. This is in line with research findings that boys tend to interact in groups where competition, and thus the number of opponents, plays an important role [47,48].

### Strengths and Limitations

This study contributes to previous studies that investigated (negative) correlates of victimization by using several indicators of victimization [20]. By taking into account the frequency and multiplicity of victimization as well as the number of bullies involved, differences in the emergence of psychological and social adjustment problems can be better understood.

It was found that the frequency of victimization is associated most with students’ psychosocial well-being and their social standing in the classroom. Nevertheless, the multiplicity of victimization and the number of bullies involved additionally contribute to the explanation of differences in psychological and social adjustment, apart from symptoms of depressiveness and popularity in the classroom. In other words, those who are often victimized, victimized in multiple ways, or victimized by more than one bully have been found to be most at risk for problems with social anxiety and well-being at school, as well as acceptance and rejection among classmates. In addition to looking at the frequency with which the victimization occurs, future research on victimization should, therefore, also investigate whether it matters by how many peers and in how many ways people are victimized.

Another strength of this study is that (assumed) less severe victimization was included, in the sense that we distinguished non-victims from victims of occasional and frequent victimization, victims of single victimization from victims of multiple victimization, and victims with one bully from victims with several bullies in the classroom. Our results clearly indicate that also students who were less severely victimized, that is, one or two times, in one way, or by one peer, were more likely to suffer from psychosocial maladjustment and a low social standing in the classroom than non-victims. However, in several previous studies of the consequences of victimization, students who indicated being victimized sometimes were considered non-victims [8,17]. The present findings illustrate that occasionally victimized students cannot necessarily be put together in a group with non-victims. Hence, in order to get a more thorough insight into the consequences of victimization, more detailed measures of victimization should be used, rather than dichotomies of victims versus non-victims.

Some limitations of this study should be considered. First, we were unable to draw causal conclusions due to the cross-sectional data. Although it appears reasonable that victimization leads to psychosocial adjustment problems [4,5] and a lower social standing in the classroom [11], the opposite may also be true. Psychologically unstable [8] and low-status children [49] might be more at risk of becoming victims.

A second limitation is the potential influence of shared method variance, given that both students’ psychosocial well-being and victimization are based on self-reports. It is, therefore, possible that the association between victimization and psychosocial well-being is inflated. Depressed or anxious children may not construe or report their victimization experiences accurately, as children who have negative feelings towards one aspect of life tend to think negatively about other aspects, too [3]. Especially regarding the measures of the number of bullies involved and the multiplicity of victimization, little is known about the validity of the self-reports. Future research should be invested in this. Nonetheless, our outcomes concerning students’ social standing in the classroom, which are based on peer reports about acceptance, rejection, and perceived popularity, suggest that the effects are unlikely to be exclusively due to fact that the indicators of victimization and psychosocial well-being are both based on self-reports.

Third, we were not able to test the attribution mechanisms directly. However, attribution theory was helpful in generating hypotheses on differences in psychosocial adjustment problems related to different aspects of victimization. Future research should investigate the considerations and feelings of victims so that it can be examined whether victims of frequent or multiple victimization, or victims with several bullies, are more likely to blame the harassment on features of the self, and, therefore, are more vulnerable to psychosocial adjustment problems. Moreover, the “distinctiveness of victims” should be taken into account. For instance, it would be interesting to examine whether victims of frequent victimization are more likely to attribute the victimization to personal characteristics when there are few other frequently victimized children in their classroom. In this way, also the sex differences concerning the associations between the multiplicity of victimization and the number of bullies, on the one hand, and depressive symptoms and well-being at school, on the other hand, might be better understood.

### Implications

The findings of the current study illustrate that differences in the maladjustment of victims can be better understood when different aspects of victimization are investigated simultaneously. Moreover, the present findings give more insight into how victimization can be measured. Our results suggest that it is highly recommended to use more detailed measures of victimization, rather than only distinguishing between non-victims and frequent victims. The tendency, both in the literature and in practice, to consider students who are occasionally victimized as non-victims raises concerns, given that this leads a group that is at higher risk for adjustment problems to be overlooked. It thus appears that the existing literature can benefit from measuring victimization in several ways. However, future researchers should investigate the validity of the various indicators more thoroughly.

Additionally, our findings reveal that a substantial part of the students were victimized by peers from other classrooms. Relatively little is known about this. Future research should, therefore, also be focused on victimization in the broader (school) context.

The results suggest that it is important to find out who is victimized, in what ways, and by how many bullies. Anti-bullying programs should not only aim to prevent and reduce victimization, but also include social-emotional monitoring so that victims and their bullies as well as (other) students with psychosocial adjustment problems can be identified at an early stage and be targeted more effectively. It may be useful for classroom teachers to receive feedback reports about the students who are often victimized or victimized in multiple ways by the same peer. The names of students who indicate having a low level of well-being at school and those who are highly disliked by their peers may also be useful information for teachers. This information may enable teachers to more effectively intervene in bullying situations.
